# Maintenance of the Quality of Coffee (*Coffea arabica* L.) in Different Packaging and Storage Locations

**DOI:** 10.1155/ijfo/5049217

**Published:** 2025-11-27

**Authors:** Claudia Patricia Gallego, Jenny Pabón, Rubén Darío Medina, Valentina Osorio

**Affiliations:** ^1^ Discipline of Quality, Colombian National Coffee Research Center (Cenicafé) – Colombian Coffee Growers Federation (CCGF), Manizales, Caldas, Colombia; ^2^ Discipline of Biometrics, Colombian National Coffee Research Center (Cenicafé) – Colombian Coffee Growers Federation (CCGF), Manizales, Caldas, Colombia

**Keywords:** coffee quality, humidity, natural fiber, polymers, vacuum, water activity

## Abstract

The preservation of sensory properties of coffee depends on the quality of the beans, storage conditions, and packaging characteristics. Currently, there is a wide range of packages that preserve bean quality during the postthreshing stages. This research determined the effects of different packaging types and storage conditions on the physical and sensory qualities of green coffee stored for 365 days. Eight packaging materials, consisting of the natural fiber–based materials fique (FN) and paper (P‐Mc) and the polyethylene (PE)‐ and polypropylene (PP)‐based materials PE‐Max, PE‐double, PE‐Multi, PP‐PVC, PE‐EVOH, and PE‐PAV, were evaluated in four locations: Manizales (Alto de Letras), Santa Marta, Chinchiná, and a cold room. The temperature and relative humidity in Alto de Letras were constant during the evaluation period, and the sensory quality expressed as the total score SCA (Specialty Coffee Association) was preserved for 365 days in the PE‐EVOH, PE‐PAV, PE‐double, and PP‐PVC packages but not in FN. In a broad sense, the SCA score was significantly affected at different storage times, regardless of the kind of packaging for the other locations. In Santa Marta, which is characterized by higher temperature and relative humidity, the SCA score of the samples was affected after 60 days, while in the cold room, the score was affected at 240 days. In Chinchiná, the SCA score decreased throughout the storage time. The environmental storage conditions affected the ability of the different kinds of packaging to preserve coffee quality. Our findings show a new alternative to maintain coffee quality from the farm and during its commercialization chain.

## 1. Introduction

Among the factors related to good practices for preserving quality during coffee processing, storage is essential because beans are exposed to variations in moisture content and water activity due to variations in the temperature and relative humidity of the environment [1]. As a result of these variations, various coffee bean characteristics that determine the physical and sensory quality of the drink begin to change; therefore, it is necessary to maintain a dry, cool environment with controlled oxygenation and isolation from direct light [2].

Because the coffee bean is composed of living cells and tissues, it undergoes physicochemical and enzymatic changes over time in response to the storage environment before the roasting process and beverage preparation. These changes affect coffee quality′s physical, chemical, and sensory components [3]. Inadequate storage conditions lead to sensory defects, such as aging defects, characterized by unpleasant woody flavors, low acidity, a weak body, and a dirty and heavy residual flavor [4]. Therefore, the storage conditions must be controlled to avoid economic losses and reductions in bean quality.

Therefore, different authors have investigated the storage characteristics with the greatest impact on the deterioration of the physical and sensory quality of the drink. Rendón et al. [5] showed that regardless of the type of packaging (conventional, nylon, polyethylene (PE), or aluminum), bean tissues deteriorate and affect quality due to variations over time in the moisture content of the coffee beans and interactions with the surrounding air. Anokye‐Bempah et al. [6], using similar package shapes, concluded that damage to coffee quality occurs over time if environmental conditions, mainly temperature and relative humidity, are variable, causing the migration of water vapor and generating hygroscopic disequilibria in the coffee beans.

In an assessment of environmental storage variables, Nasiro [7] stored coffee in different types of packaging in Ethiopia and suggested that the quality characteristics of coffee (*Coffea arabica*) can be preserved for 6 months by maintaining a storage temperature of 15°C and a bean moisture content between 17% and 22%. Anokye‐Bempah et al. [6] in California (United States) assessed the effect of hermetic packaging and jute sacks during 42 days with and without desiccant under three conditions: environmental chambers (12 days at 26°C and 75% of relative humidity), ocean transportation (18 days at 26°C and 80% of relative humidity), and land transportation (18 days at 10°C and 85% of relative humidity). Their findings indicated that hermetic packaging without a desiccant maintained adequate moisture levels in the beans. However, during transportation, the use of a desiccant in the packaging is necessary to prevent an increase in humidity that could negatively affect coffee quality. Tripetch and Borompichaichartkul [8] reported that during 15 months of storage under environmental conditions with relative humidity between 50% and 93% and temperatures of 24.5°C–27°C in Thailand, *Arabica* green coffee beans packaged in high‐density polyethylene (HDPE) and jute bags showed differing results. Specifically, they found that the coffee quality was damaged in jute bags, associated with an increase in moisture content and color variation, which induced the appearance of biochemical and enzymatic problems, altering the composition of precursors of flavor and aroma.

The same researchers reported that the quality remained slightly higher in HDPE packaging. One of the main reasons was the significant increase in moisture content, which increased from 8.0% to 14% during the first 90 days of storage for coffee packed in jute bags.

Santamaría‐Burgos [9] evaluated the germination potential of coffee seeds over a 12‐month period using different types of packaging materials: PE, fique (FN), and paper under controlled conditions of temperature (10.5°C) and relative humidity (65%). PE packaging provided the most favorable conditions for maintaining the seeds′ moisture equilibrium. However, although PE limits moisture exchange with the environment, it may also promote deterioration processes if the seeds do not have an adequate initial moisture content. This underscores the importance of considering not only the type of packaging but also the seeds′ initial physiological condition and the intended storage duration.

Both relative humidity and temperature determine the speed of metabolic activity in beans [10]. Hence, monitoring and control depend upon, among many aspects, the presence of fungi [11] whose activity and growth produce compounds that affect the quality and safety of coffee, including mycotoxins such as ochratoxin A [2, 12]. Additionally, physical quality is affected by the discoloration of the bean [6], which corresponds to flavors such as woody and earthy in the coffee drink [4], which are mainly associated with the denaturation of aroma and flavor precursor chemicals [13].

Colombian coffee production operates within diverse environmental contexts, characterized by significant variations in climatic conditions, relative humidity, and temperature. Consequently, producers require robust strategies for quality maintenance across the entire supply chain. A critical determinant of coffee storage stability is the final moisture content of the green bean, ideally maintained between 10% and 12%, irrespective of the cultivar or initial processing method. Besides, this study acknowledges the importance of chemical characterization during storage. However, our research specifically evaluated packaging types and environmental conditions. We focused on physical and sensory characteristics, as these directly determine coffee acceptability.

## 2. Materials and Methods

### 2.1. Location

The research was conducted in Colombia across four locations with contrasting temperature and relative humidity conditions: Manizales (Alto de Letras), Santa Marta, a cold room, and Chinchiná. Table 1 presents the geographical location, the altitude, the annual average temperature, and the relative humidity values for each location where green coffee was stored. Temperature and humidity values were recorded at each site throughout the evaluation period. The location at the lowest altitude was Santa Marta (18 m), while the highest was Alto de Letras in Manizales at 3.677 m above sea level. All coffee samples were stored in warehouses defined exclusively for green coffee. The warehouses in Manizales (Alto de Letras), Santa Marta, and Chinchiná had natural ventilation, concrete floors, and brick walls; instead, the cold room was a steel cabin with a hermetic and waterproof door and under controlled conditions of temperature and relative humidity. In all the localities where coffee packed was located, it was located on plastic shelves. Physical analyses of the beans and sensory tests were carried out in the Quality Laboratory in Cenicafé Plan Alto (Manizales).

**Table 1:** Locations and environmental characteristics of the experimental sites (localities).

**Localities**	**Department/municipality**	**Geographic coordinates**		**Altitude**	**Temperature (°C)**			**Relative humidity (%)**		
		**Lat**	**Long**	**m**	**Mean**	**Min**	**Max**	**Mean**	**Min**	**Max**
Alto de Letras	Caldas/Manizales	5.07° N	−75.513° W	3.677	10.3	9.1	11.9	77	70	83
Santa Marta	Magdalena/Santa Marta	11.240° N	−74.211° W	18	28.4	26.9	30.6	80	50	92
Cold room	Caldas/Manizales	4.594° N	−75.353° W	1.368	11.3	10.3	12.2	72	60	90
Naranjal Central Station	Caldas/Chinchiná	4.969° N	−75.648° W	1.420	22.6	21.2	23.9	81	65	92

### 2.2. Coffee Processing

All coffee fruits evaluated (12 ton), Castillo variety [14], were collected at Naranjal Experimental Station during the main harvest of 2021 at maturity Stages 5 and 6 according to the Cromacafé scale [15]. The coffee was classified hydraulically to remove inferior quality fruits and pulped using a calibrated horizontal cylinder machine. The pulped coffee beans underwent spontaneous fermentation, and the fermentation time was controlled at 15 h with the Fermaestro system [16]. Subsequently, the coffee was mechanically washed with Ecomill equipment [17]. The washed coffee was dried using a mechanical silo with a static layer and a drying air temperature of no more than 45°C until the beans reached a final moisture content between 10% and 12%. The dry parchment coffee was threshed in continuous flow equipment, and the resulting green coffee beans were selected by size on a sieve with a gauge of 8.4 mm (16 mesh). Broken, black, and sour beans were manually removed to obtain coffee without physical defects.

### 2.3. Coffee Packaging

**Figure 1:**
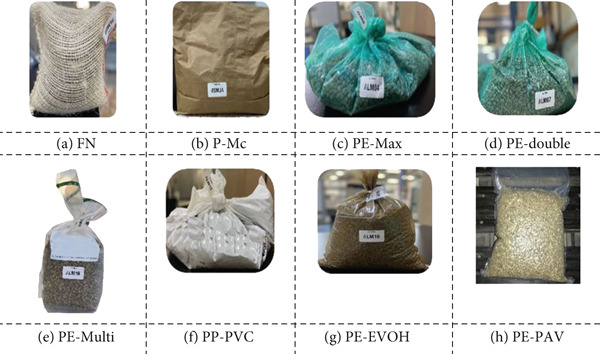
Representative images of the packages: (a) FN (fique), (b) P‐Mc (paper), (c) PE‐Max (high‐density polyethylene and polypropylene), (d) PE‐double (double barrier of high‐ and low‐linear density polyethylene), (e) PE‐Multi (high‐density polyethylene multilayer), (f) PP‐PVC (polypropylene with polyvinyl chloride), (g) PE‐EVOH (high‐density polyethylene with ethylene vinyl alcohol copolymer), and (h) PE‐PAV (low‐density polyethylene and polyamide (nylon) under vacuum).

For each site, green coffee was packaged in the following eight packages (treatments): natural fibers (no water vapor barrier), including (a) FN and (b) multilayer paper (P‐Mc), and high‐barrier polymers based on PE and polypropylene (PP) that are impermeable to gases and water vapor: (c) high‐density polyethylene and polypropylene (PE‐Max), (d) double barrier of high and low linear density polyethylene (PE‐double), (e) high‐density polyethylene multilayer (PE‐Multi), (f) polypropylene with polyvinyl chloride (PP‐PVC), (g) high‐density polyethylene with ethylene vinyl alcohol copolymer (PE‐EVOH), and (h) low‐density polyethylene and polyamide (nylon) under vacuum (PE‐PAV) (Figure 1). Each package contained 3.5 kg of green coffee beans (experimental unit). There were 96 experimental units for each site and package in order to select three units from each treatment at 60, 120, 240, and 365 days after storage, for a total of 384 units. The evaluation of response and complementary variables at time zero was performed on the selected green coffee mass at the time the units were formed.

### 2.4. Variables

#### 2.4.1. Environmental Variables

The temperature and relative humidity were recorded at each experimental location daily for 365 days using mechanical and electronic thermohygrograph equipment (Lambrecht and Testo 174, respectively).

#### 2.4.2. Physical Quality

Before storage (Time 0) and after 60, 120, 240, and 365 days of coffee storage, the physical properties of the beans were evaluated; moisture content on a wet basis (*w*_*b*_) was measured, as well as water activity (*a*_*w*_), color (CieL^∗^ *a* ^∗^ *b*), and the percentages of healthy beans (by manual visual inspection of beans without imperfections), discolored beans (by separating beans with different shades of green), insect‐damaged beans (by identifying perforations on the surface of the beans), and defective beans (broken and damaged) per experimental unit. The physical and moisture analyses were carried out in accordance with technical standards and coffee quality procedures [18]. Moisture was determined directly in duplicate. A total of 10 g of coffee was weighed into a Petri dish and placed in an oven at a temperature of 105^°^C ± 1.0^°^C for 16 h [19]. The water activity (*a*_*w*_) was determined in the coffee beans using Lab Master Neo‐Novasina equipment via a manual method with temperature control (25°C), where stable, balanced values were considered to be reached when the variation did not exceed ±0.003 for 2 min.

Color was quantitatively determined in the CIEL^∗^ *a* ^∗^ *b* ^∗^ color space with a Minolta model CR 410c colorimeter by direct reading of three coordinates: luminosity (*L* ^∗^), which measures the intensity of the reflected color with values between 0 and 100, where zero is black and 100 is white; the *a* ^∗^ coordinate, which measures the amount of green (negative) and red (positive) in the image; and the *b* ^∗^ coordinate, which measures the amount of blue (negative) and yellow (positive) in the image. The percentage of healthy beans was determined by counting the beans without any physical defects. Subsequently, the discolored kernels, insect‐damaged beans, and other defective beans were separated to estimate their respective percentages.

#### 2.4.3. Sensory Quality

Sensory analysis was conducted at 0, 60, 120, 240, and 365 days by the Cenicafé Cupping Panel, which consisted of five Q‐Grader‐certified tasters from the Coffee Quality Institute (CQI), following the SCA evaluation protocol [20]. Ten attributes of the coffee drink were evaluated: fragrance/aroma, flavor, residual flavor, acidity, body, balance, uniformity, clean cup, sweetness, and taster′s score. The sum of the scores of each attribute was calculated as the total score, which was considered the response variable.

#### 2.4.4. Experimental Design

At each evaluation site, a completely randomized experimental design with a single‐factor structure was applied, where the factor was the type of packaging. An evaluation of eight levels of this factor was conducted, corresponding to the various types of packaging utilized for grain storage. Given that the measurements for each treatment were taken repeatedly at 60, 120, 240, and 365 days, the design incorporated repeated measurements over time. This allowed for the evaluation of not only the effect of the packaging but also the changes in response and complementary variables during the storage period. For each site, treatment, and evaluation time, three experimental units were selected. This methodological approach permitted the analysis of both the primary effects and potential interactions between packaging and storage time, while accounting for the correlational structure of the measurements obtained on the same experimental unit.

#### 2.4.5. Data Analysis

For each site and evaluation time, the mean, maximum, minimum, and standard deviation of the bean characteristics were estimated.

A mixed model for repeated measures was used to analyze the SCA total score. A composite symmetric covariance structure was incorporated into this model to account for the correlation between repeated observations from the same experimental units. The model was used to evaluate the effects of packaging and time at each site.

When a significant interaction effect was shown by the analysis of variance, an analysis of the simple effects was performed using the Bonferroni multiple comparison test, which controls the family‐wise error rate. For cases with no interaction but significant main effects, the Bonferroni test was applied to the packaging treatments, while orthogonal polynomial contrasts were used to explore temporal trends (e.g., linear, quadratic, and cubic) in the response variable.

This combination of analytical techniques enabled a robust interpretation of grain behavior during storage under various packaging conditions and environmental settings.

## 3. Results and Discussion

### 3.1. Environmental Conditions

**Figure 2:**
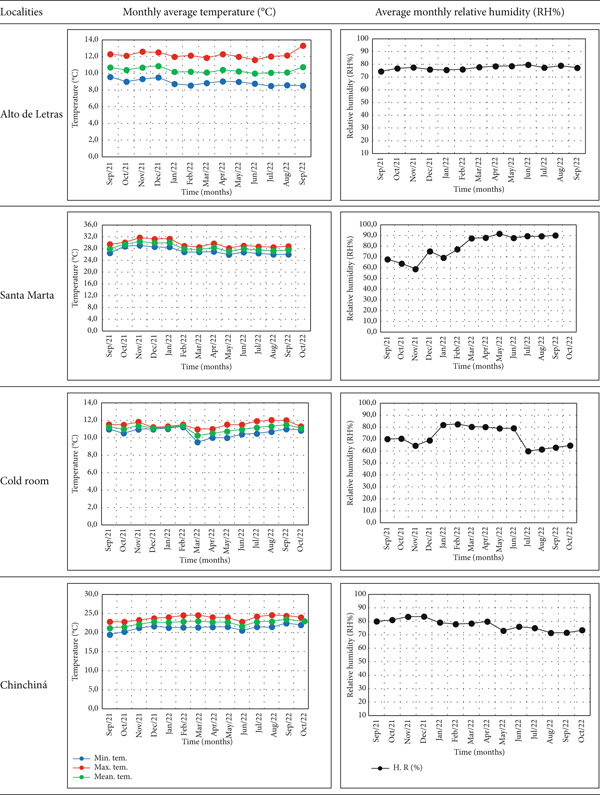
Climate variables at the four coffee storage locations.

The environmental conditions of the storage sites were tracked during the study period. The mean environmental temperature was the highest in Santa Marta (28°C), followed by Chinchiná (22.6°C), the cold room (11.3°C), and Alto de Letras (10.3°C). The relative humidity exhibited a similar trend, with average values of 80%, 81%, 77%, and 72%, respectively. The temperature remained constant at each site throughout the experiment (Figure 2). At the same time, the relative humidity exhibited greater variations, mainly in the cold room (controlled by a dehumidifier, equipment‐70P) and Santa Marta, where it tended to increase during the intermediate and final phases of the experiment. Although the physicochemical and sensory quality of coffee can be affected by various environmental factors during storage, it is common for accelerated deterioration to occur in environments with a relative humidity greater than or equal to 70% and temperatures below 10°C or higher than 20°C [21].

### 3.2. Moisture Content of Stored Green Coffee

The environmental storage conditions affected the final moisture content of the green coffee in all packaging types. The initial moisture content of the green coffee was approximately 11.8% before storage. After 365 days, the value increased to an average of 15.0% in the natural fiber packages that were permeable to oxygen and water vapor (FN and P‐Mc) in Alto de Letras, the cold room, and Chinchiná (Table 2).

**Table 2:** Average moisture content (percentage) of green coffee stored in each type of packaging.

**Locality**	**Alto de Letras** **Days**				**Santa Marta** **Days**				**Cold room** **Days**				**Chinchiná** **Days**			
**Packaging**	**60**	**120**	**240**	**365**	**60**	**120**	**240**	**365**	**60**	**120**	**240**	**365**	**60**	**120**	**240**	**365**
FN	13.42	13.52	14.46	14.81	11.42	9.92	11.97	12.47	12.38	12.04	16.58	15.17	15.20	14.72	15.50	14.16
P‐Mc	13.85	13.70	14.98	14.69	11.55	9.93	11.79	12.40	12.53	12.00	15.79	15.76	14.62	14.31	15.52	14.27
PE‐Max	11.55	11.62	11.78	12.24	11.67	11.78	11.92	12.08	11.69	11.94	11.93	12.19	11.60	11.91	12.31	12.33
PE‐Multi	11.79	11.70	11.84	12.11	11.86	11.58	11.78	12.01	11.87	11.89	11.86	11.90	11.91	11.92	12.11	12.32
PE‐EVOH	11.96	11.82	12.04	11.84	11.84	11.90	11.78	12.06	11.74	11.52	12.09	11.88	11.85	12.00	12.26	12.48
PE‐double	11.83	11.88	11.86	11.93	11.82	12.24	11.93	11.95	11.79	12.34	11.92	12.04	11.87	11.86	12.17	12.08
PP‐PVC	11.56	11.71	11.99	12.18	11.70	11.64	11.80	11.91	11.68	11.95	12.06	12.01	11.81	12.14	12.43	12.59
PE‐PAV	11.74	11.70	11.92	11.98	11.71	12.19	12.03	12.06	11.86	11.98	12.50	11.87	11.89	12.09	12.50	12.25

Abbreviations: FN, fique; PE‐double, double barrier of high‐ and low‐linear density polyethylene; PE‐EVOH, high‐density polyethylene with ethylene vinyl alcohol copolymer; PE‐Max, high‐density polyethylene and polypropylene; PE‐Multi, high‐density polyethylene multilayer; PE‐PAV, low‐density polyethylene and polyamide (nylon) under vacuum; P‐Mc, paper; PP‐PVC, polypropylene with polyvinyl chloride.

The value significantly increased with the P‐Mc packaging in cold room conditions, reaching 15.8%. These results agree with those reported by Ribeiro et al. [22]. Borém et al. [23] showed that under controlled conditions, in barrier‐free packaging such as hermetic bags, jute bags, and paper, coffee tends to absorb moisture as the storage time progresses, even in periods shorter than this study (6 months). In contrast, polymeric packaging, such as PE‐Max, PE‐double, PE‐Multi, PP‐PVC, PE‐EVOH, and PE‐PAV, maintained the moisture content of green coffee at values lower than 12.34% in Chinchiná, 12.04% in Alto de Letras, 12.01% in Santa Marta, and 11.98% in the cold room. Tripetch and Borompichaichartkul [8] showed that although packaging made of polymers with a higher protection barrier may slightly increase in bean moisture, this increase is less pronounced than the increase in moisture observed with natural fiber packaging.

Based on these findings, using PE‐Max, PE‐double, PE‐Multi, PP‐PVC, PE‐EVOH, and PE‐PAV packaging in specific environments can mitigate the deterioration of green coffee for up to 365 days. This can reduce the risk of losses in coffee bean and drink quality due to a high bean moisture content, which is associated with the presence and activity of biological agents (filamentous fungi) in bean tissues [24, 25].

### 3.3. Water Activity (*a*_*w*_)

The water activity, temperature, and moisture content of coffee strongly impact bean preservation since these parameters significantly affect chemical and microbial reactions [6, 26]. Storing beans under inadequate conditions during the first months in packages with a low protection barrier increases the risk of beans with a moisture content greater than 12% and *a*_*w*_ greater than 0.7, which increases the probability of the growth of microorganisms that alter the safety of the bean and affect the roasting process [1]. When the moisture content of the coffee beans was higher, the *a*_*w*_ of the green coffee stored in Alto de Letras, the cold room, and Chinchiná increased to an average value of 0.74 in the natural fiber packaging (FN and P‐Mc) (Table 3). These values are greater than the suitable content for preservation of the bean and increase the probability of sensory defects. Estrada‐Bahena et al. [27], using commercial coffee from jute bags, evaluated the effect of polietilene bags under desiccator conditions, reporting that after storage at 35°C for 60 days, the water activity increased to between 0.628 and 0.773. In contrast, the *a*_*w*_ values in the PE‐Max, PE‐double, PE‐Multi, PP‐PVC, PE‐EVOH, and PE‐PAV packages were less than 0.7 at the four storage locations.

**Table 3:** The average water activity (*a*_*w*_) of green coffee stored in each type of packaging.

**Locality**	**Alto de Letras** **Days**				**Santa Marta** **Days**				**Cold room** **Days**				**Chinchiná** **Days**			
**Packaging**	**60**	**120**	**240**	**365**	**60**	**120**	**240**	**365**	**60**	**120**	**240**	**365**	**60**	**120**	**240**	**365**
FN	0.68	0.67	0.73	0.73	0.62	0.54	0.65	0.66	0.64	0.59	0.77	0.74	0.74	0.73	0.76	0.74
P‐Mc	0.69	0.68	0.73	0.73	0.63	0.55	0.66	0.65	0.64	0.59	0.75	0.75	0.71	0.72	0.76	0.74
PE‐Max	0.61	0.59	0.62	0.63	0.62	0.61	0.65	0.67	0.62	0.58	0.62	0.61	0.61	0.59	0.64	0.65
PE‐Multi	0.61	0.58	0.61	0.60	0.63	0.63	0.65	0.66	0.60	0.59	0.62	0.61	0.62	0.61	0.63	0.64
PE‐EVOH	0.61	0.59	0.61	0.61	0.63	0.62	0.65	0.65	0.62	0.59	0.62	0.61	0.62	0.61	0.63	0.64
PE‐double	0.61	0.59	0.62	0.62	0.63	0.62	0.65	0.67	0.62	0.59	0.61	0.62	0.62	0.58	0.64	0.64
PP‐PVC	0.61	0.60	0.62	0.61	0.63	0.63	0.65	0.66	0.59	0.60	0.62	0.62	0.62	0.62	0.64	0.65
PE‐PAV	0.62	0.60	0.63	0.62	0.63	0.63	0.66	0.67	0.62	0.60	0.62	0.60	0.62	0.61	0.64	0.63

Abbreviations: FN, fique; PE‐double, double barrier of high‐ and low‐linear density polyethylene; PE‐EVOH, high‐density polyethylene with ethylene vinyl alcohol copolymer; PE‐Max, high‐density polyethylene and polypropylene; PE‐Multi, high‐density polyethylene multilayer; PE‐PAV, low‐density polyethylene and polyamide (nylon) under vacuum; P‐Mc, paper; PP‐PVC, polypropylene with polyvinyl chloride.

### 3.4. Color

The initial color of the coffee beans was between green and blue, a hue closely associated with coffee with a moisture content between 10% and 11.5% [28]. At lower moisture contents (< 10%), it is common for the color to appear grayish‐green as a result of excessive drying, which causes physical defects by triggering of biochemical reactions that significantly influence the appearance of beans [8]. Regarding the values of the coordinates *L* ^∗^, *a* ^∗^, and *b* ^∗^ (Tables 4, 5, and 6), color varied according to the evaluation site, treatment, and storage time, with respect to the initial evaluation Time 0 (*L* ^∗^ 72.51, *a* ^∗^ −2.33, and *b* ^∗^ 11.52). For Alto de Letras and Santa Marta, changes in the *L* ^∗^ coordinate occurred due to the effect of time, with cubic and linear behavior, respectively, according to the orthogonal contrast test (*p* < 0.0001). The results showed that at for all packaging types at Alto de Letras, the *L* ^∗^ value and therefore bean discoloration increased at 120 days and reached the maximum value at 240 days after starting the experiment. For Santa Marta, the *L* ^∗^ coordinate indicated that the luminosity (discoloration) increased with storage time in all experimental units. Regarding the cold room and Chinchiná sites, the interaction of treatment (packaging) and storage time had an effect (*p* < 0.0009).

**Table 4:** Average values and standard deviation of the color coordinate (*L* ^∗^) according to the locality, packaging, and storage time^b^.

**Locality**	**Packaging**	**Days**							
		**60**		**120**		**240**		**365**	
		**Average**	**SD** ^**a**^	**Average**	**SD**	**Average**	**SD**	**Average**	**SD**
Alto de Letras	FN	71.67	1.67	71.83	1.49	72.37	0.37	73.85	0.25
	PE‐double	73.74	1.28	73.36	0.44	75.69	0.55	74.47	1.70
	PE‐EVOH	72.64	1.81	73.53	0.90	76.20	1.77	72.42	1.24
	PE‐Max	74.47	0.58	71.95	0.67	77.54	0.67	74.19	0.37
	PE‐Multi	73.00	1.01	75.03	1.82	76.98	0.09	73.97	1.38
	PE‐PAV	73.14	1.50	73.04	2.21	75.03	1.43	72.48	1.56
	P‐Mc	70.62	2.10	72.47	2.35	74.14	1.06	72.97	2.82
	PP‐PVC	73.27	0.95	74.33	0.81	74.50	1.20	72.01	1.26

Santa Marta	FN	75.78	0.30	81.06	1.12	80.91	1.62	84.56	1.98
	PE‐double	76.30	1.06	77.58	0.91	80.49	1.70	82.38	1.24
	PE‐EVOH	76.62	1.10	78.10	1.87	81.65	2.85	82.03	0.77
	PE‐Max	75.13	1.43	78.13	1.07	83.00	1.12	83.79	1.53
	PE‐Multi	75.62	1.39	77.86	1.23	80.95	1.49	83.41	1.15
	PE‐PAV	75.15	1.36	77.23	1.85	80.06	1.14	83.01	1.51
	P‐Mc	77.63	1.51	80.40	0.95	80.50	1.26	83.80	1.48
	PP‐PVC	72.97	1.58	77.63	1.07	80.64	2.62	82.97	1.65

Cold room	FN	68.68	1.60	74.84	0.92	74.70	1.08	78.91	0.97
	PE‐double	72.00	0.72	73.42	1.01	74.11	1.70	74.09	1.50
	PE‐EVOH	72.12	1.44	73.59	1.69	74.44	1.13	74.42	1.38
	PE‐Max	72.25	1.66	75.04	0.86	73.83	0.64	74.74	2.24
	PE‐Multi	72.29	1.02	74.86	0.89	72.69	0.26	73.53	2.23
	PE‐PAV	71.53	0.91	73.57	1.72	73.31	0.72	73.86	0.40
	P‐Mc	70.85	1.08	75.25	1.10	74.21	1.63	78.22	0.59
	PP‐PVC	72.42	1.10	73.76	1.46	71.98	3.19	74.04	0.51

Chinchiná	FN	75.43	2.73	79.06	2.52	83.55	1.38	85.67	0.99
	PE‐double	74.74	1.10	72.47	4.32	76.56	1.47	77.14	0.79
	PE‐EVOH	74.03	0.64	75.92	2.36	75.81	2.00	78.59	0.28
	PE‐Max	75.03	2.52	75.78	1.64	75.41	1.92	76.48	0.21
	PE‐Multi	71.53	0.51	77.04	1.65	77.19	2.04	79.19	0.24
	PE‐PAV	74.74	0.74	74.13	0.58	75.25	1.15	77.82	0.19
	P‐Mc	73.53	0.55	79.43	2.19	83.18	2.99	86.82	0.27
	PP‐PVC	73.74	0.91	77.05	1.30	76.09	2.52	78.17	0.49

^a^Standard deviation.

^b^ *L* value coordinate at Time 0: 72.51.

**Table 5:** Average values and standard deviation of the color coordinate (*a* ^∗^) according to the locality, packaging, and storage time^b^.

**Locality**	**Packaging**	**Days**							
		**60**		**120**		**240**		**365**	
		**Average**	**SD** ^**a**^	**Average**	**SD**	**Average**	**SD**	**Average**	**SD**
Alto de Letras	FN	−2.39	0.12	−2.16	0.10	−2.55	0.13	−2.39	0.04
	PE‐double	−2.56	0.19	−2.49	0.10	−2.43	0.15	−2.51	0.21
	PE‐EVOH	−2.46	0.10	−2.37	0.16	−2.41	0.27	−2.27	0.12
	PE‐Max	−2.38	0.05	−2.42	0.04	−2.38	0.08	−2.36	0.22
	PE‐Multi	−2.47	0.10	−2.62	0.20	−2.33	0.10	−2.26	0.10
	PE‐PAV	−2.58	0.19	−2.37	0.17	−2.23	0.12	−2.53	0.23
	P‐Mc	−2.29	0.13	−2.14	0.13	−2.65	0.10	−2.45	0.14
	PP‐PVC	−2.50	0.06	−2.50	0.15	−2.49	0.28	−2.01	0.10

Santa Marta	FN	−2.60	0.14	−2.37	0.21	−1.99	0.08	−1.63	0.16
	PE‐double	−2.53	0.05	−2.38	0.17	−2.17	0.18	−1.42	0.06
	PE‐EVOH	−2.64	0.12	−2.41	0.14	−2.36	0.14	−1.72	0.16
	PE‐Max	−2.53	0.20	−2.26	0.12	−2.05	0.25	−1.70	0.08
	PE‐Multi	−2.79	0.10	−2.34	0.21	−2.25	0.54	−1.74	0.39
	PE‐PAV	−2.70	0.05	−2.41	0.15	−1.99	0.57	−1.81	0.06
	P‐Mc	−2.76	0.33	−2.42	0.11	−1.81	0.35	−1.26	0.19
	PP‐PVC	−2.48	0.07	−2.31	0.15	−2.10	0.18	−1.78	0.13

Cold room	FN	−2.22	0.19	−2.51	0.19	−2.33	0.18	−2.78	0.20
	PE‐double	−2.53	0.21	−2.37	0.10	−2.59	0.20	−2.67	0.11
	PE‐EVOH	−2.59	0.01	−2.40	0.13	−2.55	0.16	−2.58	0.14
	PE‐Max	−2.68	0.13	−2.61	0.20	−2.49	0.05	−2.59	0.08
	PE‐Multi	−2.49	0.23	−2.44	0.09	−2.42	0.19	−2.64	0.09
	PE‐PAV	−2.45	0.13	−2.34	0.24	−2.54	0.05	−2.73	0.07
	P‐Mc	−2.51	0.08	−2.55	0.06	−2.56	0.20	−2.90	0.27
	PP‐PVC	−2.85	0.13	−2.40	0.06	−2.50	0.22	−2.58	0.08

Chinchiná	FN	−2.37	0.32	−2.45	0.14	−2.17	0.10	−2.12	0.19
	PE‐double	−2.74	0.13	−2.17	0.55	−2.55	0.21	−2.55	0.08
	PE‐EVOH	−2.69	0.19	−2.32	0.41	−2.36	0.37	−2.65	0.30
	PE‐Max	−2.67	0.07	−2.40	0.15	−2.36	0.17	−2.61	0.09
	PE‐Multi	−2.43	0.13	−2.26	0.18	−2.65	0.18	−2.64	0.09
	PE‐PAV	−2.71	0.10	−2.25	0.22	−2.39	0.38	−2.50	0.02
	P‐Mc	−2.38	0.27	−2.31	0.21	−2.43	0.17	−2.22	0.19
	PP‐PVC	−2.43	0.12	−2.22	0.17	−2.57	0.39	−2.70	0.21

^a^Standard deviation.

^b^ *a* value coordinate at Time 0: −2.33.

**Table 6:** Average values and standard deviation of the color coordinate (*b* ^∗^) according to the locality, packaging, and storage time^b^.

**Locality**	**Packaging**	**Days**							
		**60**		**120**		**240**		**365**	
		**Average**	**SD** ^**a**^	**Average**	**SD**	**Average**	**SD**	**Average**	**SD**
Alto de Letras	FN	11.44	0.33	11.08	0.27	11.49	0.54	12.15	0.40
	PE‐double	11.99	0.59	12.05	0.59	12.54	0.16	12.44	0.56
	PE‐EVOH	11.75	0.58	12.09	0.33	12.98	0.34	12.06	0.36
	PE‐Max	12.11	0.35	11.60	0.23	13.12	0.39	12.12	0.03
	PE‐Multi	11.98	0.34	12.34	0.92	12.91	0.09	12.17	0.76
	PE‐PAV	12.14	0.57	11.85	0.66	12.62	0.39	12.07	0.51
	P‐Mc	11.20	0.32	11.27	0.51	12.07	0.33	12.13	0.49
	PP‐PVC	12.00	0.43	12.03	0.52	12.38	0.53	11.71	0.31

Santa Marta	FN	13.33	0.35	15.18	0.53	15.30	0.66	18.74	0.90
	PE‐double	12.98	0.09	13.83	0.04	15.62	0.70	18.52	0.74
	PE‐EVOH	13.25	0.57	14.06	0.35	16.49	0.96	17.62	0.14
	PE‐Max	12.80	0.57	13.92	0.24	16.59	0.64	18.67	0.97
	PE‐Multi	13.08	0.30	13.95	0.67	16.01	0.24	18.15	0.92
	PE‐PAV	13.04	0.52	14.00	0.68	15.79	0.89	18.23	0.39
	P‐Mc	13.71	0.39	14.83	0.54	16.04	0.80	19.33	0.95
	PP‐PVC	12.23	0.47	13.87	0.23	16.29	0.52	18.39	0.62

Cold room	FN	10.81	0.44	12.11	0.22	11.95	0.43	13.57	0.39
	PE‐double	11.73	0.37	11.97	0.30	12.51	0.58	12.56	0.15
	PE‐EVOH	11.89	0.44	12.12	0.33	12.68	0.20	12.81	0.36
	PE‐Max	12.20	0.52	12.34	0.37	12.75	0.29	12.99	0.73
	PE‐Multi	11.89	0.27	12.27	0.23	12.14	0.06	12.67	0.38
	PE‐PAV	11.59	0.19	11.79	0.47	12.45	0.34	12.48	0.30
	P‐Mc	11.67	0.42	12.20	0.26	12.30	0.11	13.57	0.11
	PP‐PVC	12.25	0.26	12.08	0.11	12.14	0.85	12.65	0.45

Chinchiná	FN	12.11	0.59	13.91	1.01	16.33	0.33	18.65	0.52
	PE‐double	12.64	0.28	12.71	0.39	13.28	0.31	14.05	0.47
	PE‐EVOH	12.45	0.10	12.45	0.56	13.03	0.74	14.38	0.28
	PE‐Max	12.58	0.51	12.58	0.40	13.15	0.30	13.57	0.42
	PE‐Multi	11.87	0.19	12.81	0.44	13.53	0.38	14.55	0.21
	PE‐PAV	12.48	0.43	12.19	0.16	12.91	0.18	14.09	0.87
	P‐Mc	11.74	0.44	13.50	0.49	16.01	1.13	18.49	0.18
	PP‐PVC	11.96	0.37	12.89	0.14	13.39	0.82	14.10	0.44

^a^Standard deviation.

^b^ *b* value coordinate at Time 0: 11.52.

In cold room conditions, greater discoloration (increased *L* ^∗^ coordinate) occurred in coffee stored in natural fiber packaging (FN and P‐Mc) after 365 days (final evaluation time), according to Bonferroni′s multiple comparison test (*p* < 0.0009). In Chinchiná, similar behavior was observed for the natural fiber packaging after 240 days. In contrast, the values of the *L* ^∗^ coordinate remained constant in Chinchiná during the evaluation period (Bonferroni′s test *p* > 0.005), particularly in the coffee packed in the polymeric materials.

For all sites, the analysis of variance indicated that only time had an effect (*p* < 0.0001). In particular, the storage time had a cubic relationship with the *a* ^∗^ coordinate (*p* < 0.0017), indicating a general tendency toward less negative values (tending to zero) over time (Table 5). Finally, the *b* ^∗^ coordinate values were in the yellow range and exhibited a similar trend to the *L* ^∗^ coordinate values for all sites and packages (Table 6). In the case of Chinchiná, in the natural fiber packaging (FN and P‐Mc), rapid changes in the *L* ^∗^ coordinate occurred from 120 to 240 days after the experiment began.

The changes in the color of the coffee beans during storage, based on the analysis of the *a* ^∗^ and *b* ^∗^ coordinates, indicate oxidative processes and biochemical degradation. These processes have a marked influence on the precursor compounds in the bean responsible for the taste of the coffee drink and generally correspond to shades from yellow–green to light yellow [24, 29]. The results show that the *L* ^∗^, *a* ^∗^, and *b* ^∗^ coordinates can reliably indicate variations in the physical quality of coffee beans according to loss of color under the conditions, treatments, and storage times considered.

### 3.5. Physical Quality of the Bean

At the beginning of the experimental period (Day 0), the percentage of healthy beans in all treatments was 93.9%. After 365 days in the natural fiber packaging (FN and P‐Mc) in the cold room, the percentage decreased to 91.8%. Then, 89.34% were recorded in Alto de Letras and 89.5% in Santa Marta, while in Chinchiná, a greater reduction (> 85.28%) in healthy beans was observed in the FN and P‐Mc packages.

**Figure 3:**
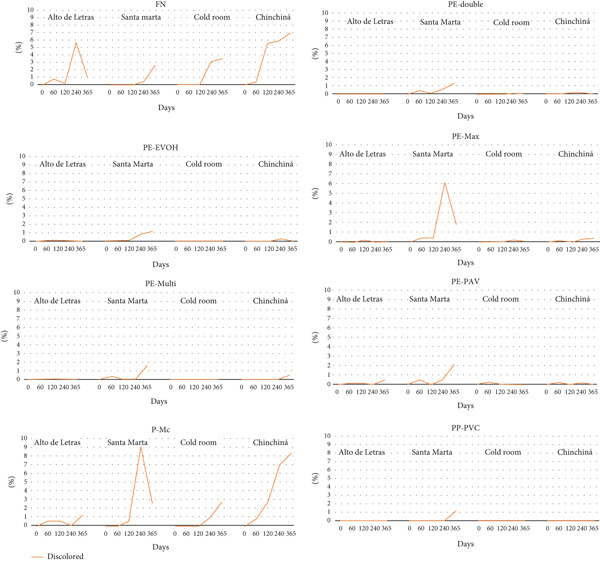
Average number of discolored beans by treatment, storage time, and location.

Bleached beans, which are an indicator of loss of physical quality, were detected at a higher percentage in the coffee stored in FN and P‐Mc in Alto de Letras, Chinchiná, and Santa Marta, with average values of 7.6%, 7.9%, and 9.05%, respectively. In Santa Marta, the highest level of discoloration (7.2%) was detected in the PE‐Max packaging (Figure 3). Bean discoloration is studied in storage scenarios due to deficiencies in the regulation of light and loss of the characteristic coloration of beans [30]. This phenomenon can be exacerbated as the storage time progresses due to moisture content, which causes oxidative reactions and enzymatic biochemical transformations, including the Maillard process, which affects the drink′s taste. This set of reactions is usually catalyzed by various factors, including moisture content [8]. This mechanism would explain the presence of discoloration in the FN and P‐Mc packages in three of the four study locations.

For the PP‐PVC, PE‐double, PE‐EVOH, PE‐Multi, and PE‐PAV packages, there were no discolored beans throughout the evaluation time, except in Santa Marta, where levels of up to 1.5% were found, regardless of the type of synthetic packaging used. These results contrast with those of Coelho et al. [31] who reported early deterioration of coffee associated with bean discoloration when hermetic plastic, aluminum, and tow bags were used as packaging. Therefore, bean quality in terms of characteristic color can be maintained depending on the location and type of packaging, such as those considered in this study.

Finally, regarding physical damage, 3.2% of the beans exhibited defects related to the coffee berry borer at the beginning of the experiment. These levels were consistent throughout the evaluations; however, at the end of the experimental period, an increase in the levels at 365 days was observed due to infestation by *Araecerus fasciculatus* (weevil) in the coffee stored in FN in Chinchiná. This observation highlights the susceptibility of coffee stored in FN to biochemical, physical, and sensory deterioration.

### 3.6. Sensory Quality

**Figure 4:**
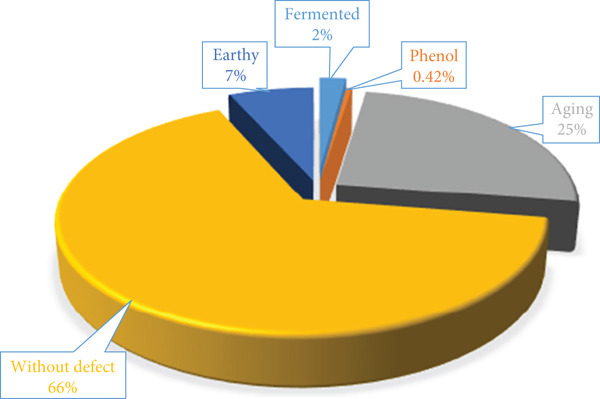
Percentage of samples without sensory defects and with various types of defects in stored coffee.

Before packaging, the green coffee obtained an initial total score of 82.5 on the SCA scale. According to the grading scale, this classifies it as a very good coffee, with no sensory defects and of a special type. The results of the sensory analysis revealed that 66% of the 384 samples analyzed during storage (365 days) had no sensory defects and maintained an average score of 81 ± 0.5 points on the SCA scale (Figure 4). However, 34% of the samples had sensory defects (25% aging, 7% earthy, 2% fermented, and 0.42% phenolic) which caused alterations in the attributes of fragrance, aroma, flavor, and acidity in the four storage locations. Aging sensory defects are characteristic of coffee stored for long periods under conditions without humidity or temperature control [32]. Furthermore, these results are consistent with those of Gallego and Rodríguez‐Valencia [4], who reported that coffee stored under variable humidity and temperature conditions for 120 days showed significant changes in sensory analysis scores associated with the appearance of aging defects and a general loss of quality.

**Figure 5:**
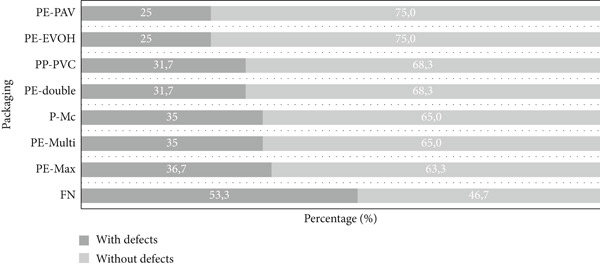
Percentage of samples with and without sensory defects by type of packaging. PE‐PAV, low‐density polyethylene and polyamide (nylon) under vacuum; PE‐EVOH, high‐density polyethylene with ethylene vinyl alcohol copolymer; PP‐PVC, polypropylene with polyvinyl chloride; PE‐double, double barrier of high‐ and low‐linear density polyethylene; P‐Mc, paper; PE‐Multi, high‐density polyethylene multilayer; PE‐Max, high‐density polyethylene and polypropylene; FN, fique.

Regarding the packaging types used in storage, the proportions of samples without and with defects differed for the different packaging materials (natural fibers and polymeric fibers with a high water vapor barrier). According to the chi‐square homogeneity test (*p* > 0.0384), the packaging associated with the most significant number of samples with sensory defects was FN (53.3%), followed by PE‐Max, P‐Mc, PE‐Multi, PE‐double, and PP‐PVC, with the lowest percentages for PE‐EVOH and PE‐PAV (Figure 5). These results confirm the findings of Borém et al. [23], who established that high‐barrier polymeric packaging provides green coffee beans with equal or superior protection to vacuum packaging.

At the end of the evaluations (365 days of storage), the sensory quality according to the total SCA score varied according to the site and the type of packaging used. In Alto de Letras, the analysis of variance showed an effect of the interaction of storage time and type of packaging (Pr > *F* = <0.0001), which means that the differences between the packages are not maintained over time, so it was pertinent to analyze simple effects. The coffee packed in PP‐PVC, PE‐PAV, PE‐EVOH, and PE‐double maintained sensory quality until the last date of measurement (365 days), while for the coffee packed in FN, P‐Mc, PE‐Max, and PE‐Multi, the sensory quality was maintained for 240 days, with the lowest total SCA score among the other packages according to Bonferroni′s multiple comparison test at 5% (Table 7).

**Table 7:** Average SCA score for various combinations of packaging and storage time in Alto de Letras.

**Storage time (days)**	**Average total score** ^**a**^ **(SCA)**															
	**FN**		**P-Mc**		**PE-EVOH**		**PE-Max**		**PE-Multi**		**PE-PAV**		**PE-double**		**PP-PVC**	
0	81.9	a	81.9	a	81.9	a	81.9	a	81.9	a	81.9	a	81.9	a	81.9	a
60	81.0	a	80.9	a	81.4	a	82.7	a	81.8	a	80.6	a	82.3	a	80.9	a
120	80.7	a	81.5	a	80.8	a	81.9	a	80.5	a	80.3	a	80.6	a	80.6	a
240	80.5	a	80.6	a	81.1	a	81.6	a	81.1	a	80.4	a	81.9	a	80.7	a
365	53.4	b	54.2	b	81.4	a	54.0	b	63.1	b	81.4	a	78.1	a	81.4	a

^a^Different letters indicate significant differences among packages for each evaluation time according to Bonferroni′s multiple comparison test at 5% and adjust the *p* values for each comparison for the time effect (more than two time levels).

**Figure 6:**
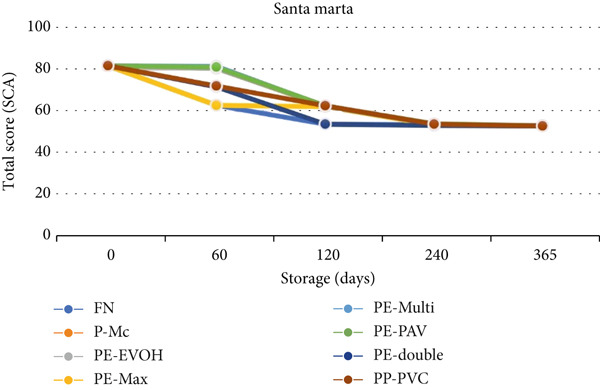
Average total SCA score of packages at different storage times, Santa Marta. FN, fique; P‐Mc, paper; PE‐EVOH, high‐density polyethylene with ethylene vinyl alcohol copolymer; PE‐Max, high‐density polyethylene and polypropylene; PE‐Multi, high‐density polyethylene multilayer; PE‐PAV, low‐density polyethylene and polyamide (nylon) under vacuum; PE‐double, double barrier of high‐ and low‐linear density polyethylene; PP‐PVC, polypropylene with polyvinyl chloride.

In Santa Marta, there was no effect of packaging, but there was an effect of storage time; that is, a deterioration in sensory quality (SCA score) occurred after 60 days of storage, regardless of the packaging. This behavior was described by an orthogonal polynomial equation (Figure 6), and the quality preservation time exhibited a quadratic or second‐order trend (Pr > *F* = 0.0006).

**Figure 7:**
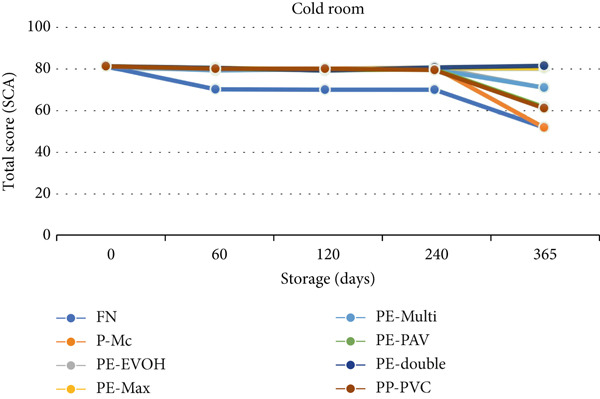
Average total SCA score of packages stored for different times in the cold room. FN, fique; P‐Mc, paper; PE‐EVOH, high‐density polyethylene with ethylene vinyl alcohol copolymer; PE‐Max, high‐density polyethylene and polypropylene; PE‐Multi, high‐density polyethylene multilayer; PE‐PAV, low‐density polyethylene and polyamide (nylon) under vacuum; PE‐double, double barrier of high‐ and low‐linear density polyethylene; PP‐PVC, polypropylene with polyvinyl chloride.

For the cold room, the analysis of variance did not reveal an interaction effect between storage time and packaging type. Under an average temperature of 11.3°C (maximum of 12.3°C and minimum of 10.3°C) and average relative humidity of 72%, regardless of the packaging material (natural fibers and polymeric high barrier), the SCA score was preserved until 240 days of storage, with a third‐degree polynomial trend (Pr > *F* = 0.0013) (Figure 7). However, the coffee stored in the PE‐Max, PE‐double, and PE‐EVOH packages maintained its initial quality until 365 days according to Bonferroni′s adjusted test. The natural fiber packages (FN and P‐Mc) showed a decrease in quality score compared to those of the three plastic packages at 365 days (Table 8).

**Table 8:** Results of the Bonferroni‐adjusted probability test comparing the PE‐Max, PE‐double, and PE‐EVOH packages to the FN package at a storage time of 365 days.

**Storage time (days)**	**Packaging**		**Adj** **p** **Bonferroni**
365	PE‐Max	FN	0.0079
365	PE‐double	FN	0.0067
365	PE‐EVOH	FN	0.0036

**Figure 8:**
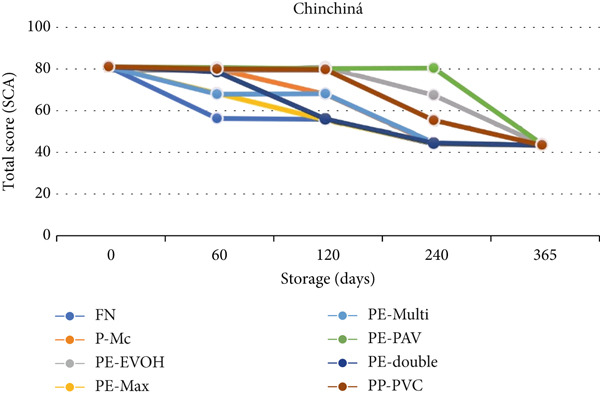
Average SCA total score for different packages at different storage times, Chinchiná. FN, fique; P‐Mc, paper; PE‐EVOH, high‐density polyethylene with ethylene vinyl alcohol copolymer; PE‐Max, high‐density polyethylene and polypropylene; PE‐Multi, high‐density polyethylene multilayer; PE‐PAV, low‐density polyethylene and polyamide (nylon) under vacuum; PE‐double, double barrier of high‐ and low‐linear density polyethylene; PP‐PVC, polypropylene with polyvinyl chloride.

Finally, for Chinchiná, the analysis of variance revealed no interaction effect between storage time and packaging, but it did show separate effects of time and packaging. The total SCA score of the FN packaging was lower than that of the PE‐PAV packaging, according to Bonferroni′s adjusted multiple comparison test (*p* = 0.0049). The effect of time presented a decreasing linear trend (*p* < 0.0001) according to the orthogonal polynomial test, indicating a deterioration of quality began to appear after 60 days of storage, especially for the natural fiber (FN) packaging (Figure 8).

## 4. Conclusions

The variations in coffee quality over time were explained by the prevailing environmental conditions at the storage site. Considering the wide diversity of environmental conditions of Colombian coffee growing, our findings indicate that the packaging natural fiber (FN and P‐Mc) could be adequate to preserve the coffee quality during short periods (< 60 days stored), while the polymeric packaging such as PE‐EVOH, PP‐PVC, PE‐PAV, PE‐Max and PE‐double maintain the coffee quality until 365 days specifically under constant environmental conditions of temperature (10°C–11°C) and relative moisture between (72%–77%).

Under the last consideration, a technical assessment is provided on the efficiency of various commercially available packaging types and their impact on the physical and sensory attributes of coffee, contributing to the preservation of its cup quality.

## Disclosure

All authors have read and accepted the published version of the manuscript.

## Conflicts of Interest

The authors declare no conflicts of interest.

## Author Contributions

Conceptualization, data curation, and writing the original draft: C.P.G. Formal analysis, methodology, and validation: C.P.G. and V.O. Drafting—review and editing: J.P., V.O., and R.D.M.

## Funding

This study was funded by the Centro Nacional de Investigaciones de Café (10.13039/100019597) (CAL103004).

## Data Availability

The data that support the findings of this study are available from the corresponding author upon reasonable request.
